# Plant functional traits differ in adaptability and are predicted to be differentially affected by climate change

**DOI:** 10.1002/ece3.5890

**Published:** 2019-11-28

**Authors:** Collin W. Ahrens, Margaret E. Andrew, Richard A. Mazanec, Katinka X. Ruthrof, Anthea Challis, Giles Hardy, Margaret Byrne, David T. Tissue, Paul D. Rymer

**Affiliations:** ^1^ Hawkesbury Institute for the Environment Western Sydney University Penrith NSW Australia; ^2^ Environmental & Conservation Sciences Murdoch University Murdoch WA Australia; ^3^ Biodiversity and Conservation Science Western Australian Department of Biodiversity, Conservation and Attractions Kensington WA Australia; ^4^ Centre for Phytophthora Science and Management Environmental & Conservation Sciences Murdoch University Murdoch WA Australia

**Keywords:** climate adaptation, *Corymbia calophylla*, general additive models, heritability, intraspecific variation, trait coordination

## Abstract

1. Climate change is testing the resilience of forests worldwide pushing physiological tolerance to climatic extremes. Plant functional traits have been shown to be adapted to climate and have evolved patterns of trait correlations (similar patterns of distribution) and coordinations (mechanistic trade‐off). We predicted that traits would differentiate between populations associated with climatic gradients, suggestive of adaptive variation, and correlated traits would adapt to future climate scenarios in similar ways.
2. We measured genetically determined trait variation and described patterns of correlation for seven traits: photochemical reflectance index (PRI), normalized difference vegetation index (NDVI), leaf size (LS), specific leaf area (SLA), δ^13^C (integrated water‐use efficiency, WUE), nitrogen concentration (N_CONC_), and wood density (WD). All measures were conducted in an experimental plantation on 960 trees sourced from 12 populations of a key forest canopy species in southwestern Australia.
3. Significant differences were found between populations for all traits. Narrow‐sense heritability was significant for five traits (0.15–0.21), indicating that natural selection can drive differentiation; however, SLA (0.08) and PRI (0.11) were not significantly heritable. Generalized additive models predicted trait values across the landscape for current and future climatic conditions (>90% variance). The percent change differed markedly among traits between current and future predictions (differing as little as 1.5% (δ^13^C) or as much as 30% (PRI)). Some trait correlations were predicted to break down in the future (SLA:N_CONC_, δ^13^C:PRI, and N_CONC_:WD).
4. Synthesis: Our results suggest that traits have contrasting genotypic patterns and will be subjected to different climate selection pressures, which may lower the working optimum for functional traits. Further, traits are independently associated with different climate factors, indicating that some trait correlations may be disrupted in the future. Genetic constraints and trait correlations may limit the ability for functional traits to adapt to climate change.

## Graphical abstract

We tested whether functional traits of an important tree species in southwestern Australia were genetically determined and able to undergo selection. Our results suggest that some traits are heritable (Water Use Efficiency) while others are not (SLA), indicating that in the future, under different climates, selection will differentially effect traits. Therefore, it is likely that current trait combinations will change with climate.

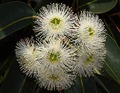

## 1 INTRODUCTION

Forests are under pressure from climate change (Bonan, 2008; Canadell & Raupach, 2008; Chazdon, 2008; Riitters et al., 2002), and impacts are expected to reduce long‐term resilience and function (Chazdon, 2008). Many organisms have evolved traits suited to their environment through the long‐term process of natural selection resulting in local adaptation (Kawecki & Ebert, 2004). However, changes to climate might negatively impact those long‐established patterns of local adaptation (Aitken & Whitlock, 2013; Hoffmann & Sgrò, 2011). While it may be many years until the full effects of global climate change are realized, the effects on local forest populations have already been observed (Harris et al., 2018; Hoffmann et al., 2019). For example, climate change impacts include changes in forest distribution (Kelly & Goulden, 2008; Lenoir et al., 2010) and widespread tree mortality due to increased severity of drought and heat waves (Allen et al., 2010; Matusick et al., 2018; Williams & Dumroese, 2013). The range and complexity of impacts of climate change on forests are likely to disrupt current patterns of adaptation, making it critical to understand the adaptive capacity of natural forests, and associated ecological and evolutionary constraints.

The adaptive capacity of trees may facilitate the long‐term persistence of natural forests. It is predicted that to account for climate change, many temperate tree species will have to migrate toward the poles or higher elevations (Aitken, Yeaman, Holliday, Wang, & Curtis‐McLane, 2008), although this pattern is not ubiquitous (see Crimmins, Dobrowski, Greenberg, Abatzoglou, and Mynsberge (2011), e.g, of downward shift in species’ optimum elevation due to changes in water balance). Evidence suggests that species migration is not occurring or has been limited due to hard boundaries such as oceans or human‐made barriers (Corlett & Westcott, 2013; Parmesan & Yohe, 2003; Zhu, Woodall, & Clark, 2012). However, many tree species show high levels of genetic variation and may have enough standing genetic variation for positive selection to occur to better match phenotype and future climate (Barrett & Schluter, 2008).

Functional traits of tree species are indicative of patterns of adaptation to their environment (Reich, 2014), and the relationship between climate and some traits is well established (Wright et al., 2005). Functional traits can reflect plant performance, stress, and allocation and therefore are shaped by selective pressures as demonstrated by trait variation along climatic gradients indicative of genetic adaptation (Reich et al., 2003). To date, there has been a focus on species mean trait values; however, for species to be able to adapt to climate change they require, heritable, intraspecific trait variation. Yet, a few studies focus on intraspecific trait variation and if these traits are genetically determined (e.g., Aranda et al., 2014; Schreiber, Hacke, & Hamann, 2015; Hajek, Kurjak, von Wühlisch, Delzon, & Schuldt,^2016^; Madani et al., 2018). There is growing appreciation of the importance of intraspecific variation in functional and complex traits in providing the capacity to adapt to climate change.

Functional traits, by definition, are indicative of their relationship to the environment (Shipley et al., 2016), and population differentiation along climate gradients can be used to quantify the relative contribution of climate variables to patterns of trait differentiation (Madani et al., 2018). But these outputs would be unable to estimate the relative trait responsiveness to selection pressures. Estimating narrow‐sense heritability is one way to calculate how much trait variation is due to genetics and estimate relative contribution of natural selection on trait differentiation (Geber & Griffen, 2003), allowing us to predict how traits may respond to new climate pressures through natural selection and genetic constraints. Together, trait heritability and climate gradients can help us predict how traits might individually and collectively evolve (or not) in the future.

The coordination of functional traits has been well studied (Reich, 2014; Wright, Falster, Pickup, & Westoby, 2006) and has been shown to be strongly linked to climate (Li et al., 2018; Mencuccini, Minunno, Salmon, Martínez‐Vilalta, & Hölttä, 2015; Reich, 2014). The worldwide leaf economic spectrum (LES) consistently explains the complex relationship between environment and leaf traits and also coordination between leaf structure and function (Donovan, Maherali, Caruso, Huber, & de Kroon, 2011; Reich, Walters, & Ellsworth, 1997; Wright et al., 2004). Specifically, it is expected that thinner leaves (high specific leaf area values) would be adapted to cooler, wetter conditions and this pattern is coordinated with high nitrogen concentrations (Wright et al., 2004). In addition, traits can be correlated across a species’ distribution, even if no mechanistic coordination is present (e.g., Wright et al., 2007). Relationships among ecologically important plant traits may be an adaptive signature from natural selection (Westoby, Falster, Moles, Vesk, & Wright, 2002). Yet the genetic basis for functional trait variation is largely unknown, including the linkage among correlated or coordinated traits and their adaptive capacity to respond to climate change.

Genetic variation in plant traits within species is essential for them to adapt to novel climate conditions by influencing establishment, survival, and fitness (Violle et al., 2007). Importantly, standing genetic variation will be required for traits to keep pace with selection imposed through processes of natural selection, enabling a species to occupy a broad range of climates and adapt to novel climate conditions (Alberto et al., 2013). Therefore, measuring variation in functional traits, estimating their heritability, and identifying possible agents of selection in a foundation tree can provide critical information in how a species might continue to persist in a changing climate. Here, we use an experimental multipopulation plantation to assess trait variation in the foundational forest canopy species, *Corymbia calophylla* R. Br. K.D. Hill and L.A.S. Johnson (*Eucalyptus* sensu lato; family Myrtaceae)*,* located in southwestern Australia. Measuring traits in an experimental plantation provides a common environment allowing for the isolation of genetic effects associated with phenotypic differences among populations. We expect that trait variation among populations will show unique patterns of adaptation associated with their climate‐of‐origin. While some traits will be heritable and show strong shifts along climate gradients (e.g., water‐use efficiency), other traits will have greater variation within and among populations, resulting in lower levels of heritability (e.g., SLA). We test the hypothesis that traits will be unequally affected by climate change, such that traits with higher levels of heritability will need to adapt to their new climates or migrate with their optimum climates. Finally, we ask if trait correlation and coordination, such as those within the LES paradigm, will maintain similar relationships in the future. We discuss the implications for the capacity to adapt to climate change and the ability to predict the coevolutionary trajectories of functional traits.

## 2 MATERIALS AND METHODS

### 2.1 Study species

*Corymbia calophylla* is a foundation forest canopy species located in Western Australia (WA). It is considered a foundation species because its characteristics are critical for forest structure and ecological processes (Ellison et al., 2005). This species is an ideal candidate in which to study adaptation of functional traits because its distribution traverses strong environmental gradients over short distances, it has recently experienced mortality events attributed to climate change (Matusick, Ruthrof, Brouwers, Dell, & Hardy, 2013; Ruthrof, Matusick, & Hardy, 2015), and evidence of adaptation to climate has been identified in physiological experiments and genome–environment investigations (Ahrens, Byrne, & Rymer, 2019; Ahrens, Mazanec, et al., 2019; Aspinwall et al., 2017; Blackman, Aspinwall, Tissue, & Rymer, 2017).

### 2.2 Experimental site and population sampling

**Figure 1:**
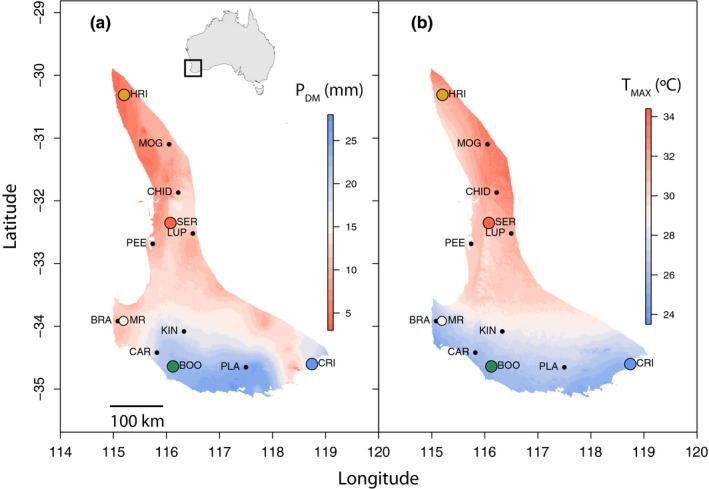
Distribution of *Corymbia calophylla* in southwestern Australia, and location of 12 populations overlaid on maps of (a) precipitation of the driest month (*P*_DM_) (mm), and (b) average maximum temperature of the warmest month (T_MAX_) (°C). The experimental planting site is denoted by the white point and labeled MR. The populations used for data set 1 are denoted by four colors for the four populations (BOO = Boorara, cool–wet climate; CRI = Cape Riche, cool–dry climate; HRI = Hill River, hot–dry climate; SER = Serpentine, hot–wet climate), and all 12 populations (colored and black points) are used for data set 2

This research was conducted in a plantation near Margaret River, WA (Figure 1), located in the cool–wet region of the distribution of *C. calophylla*. Seed collection and trial design are described in detail in Ahrens, Mazanec, et al. (2019). Briefly, 18 populations represented by 165 families were established at the experimental site for a total of 3,960 individuals in six replicated blocks. Families are defined here as individuals that have a known, common mother but an unknown father (i.e., half‐sibs). We developed two separate data sets: the first was used to estimate trait heritability, while the second was used to explore correlation and coordination among traits and its association with climate‐of‐origin. For the first data set, we focused on four populations representing four contrasting climate combinations covering the full geographic distribution of *C. calophylla* (BOO, cool–wet climate; CRI, cool–dry climate; HRI, hot–dry climate; SER, hot–wet climate; Figure 1). A total of 40 families (when available) from the four populations were sampled, with 12 replicate trees from each family, to provide estimates of phenotypic variance within and among families (480 trees). For the second data set, we collected data from 12 populations with four replicates from each of 10 families (when available) to estimate variance within and among populations (480 trees; Table 1; Figure 1).

**Table 1:** The locations and climate‐of‐origin of each population within the study along with the total number of samples for each data set

Populations	Latitude	Longitude	*T*_MAX_ (°C)	*P*_MA_ (mm)	$\frac{1}{\text{AI}}$	*P*_DM_ (mm)	Data set 1		Data set 2	
							Families	Total	Families	Total
Warm, dry climate										
HRI	−30.3114	115.2016	31.7	563	2.56	4	9	110	9	36
MOG	−31.0986	116.0509	33.3	579	2.56	10			10	40
LUP	−32.5207	116.4990	31.6	635	2.22	12			9	40
Warm, wet climate										
SER	−32.3527	116.0764	30.5	1,173	1.12	12	11	123	11	44
CHID	−31.8682	116.2229	32.2	900	1.54	12			10	40
PEE	−32.6846	115.7427	30.4	885	1.49	10			8	40
Cool, dry climate										
CRI	−34.6015	118.7427	26.2	579	2.08	20	8	99	8	32
KIN	−34.0812	116.3304	27.7	820	1.49	19			10	40
PLA	−34.6534	117.4991	26.7	733	1.59	25			10	40
Cool, wet climate										
BOO	−34.6389	116.1238	25.6	1,159	0.95	24	11	136	10	40
CAR	−34.4196	115.8213	25.9	1,106	1.02	20			10	40
BRA	−33.9164	115.0833	26.1	1,072	1.04	11			9	40
Overall							39	468	114	472

Note

The four‐population data set (data set 1) was used to estimate heritability, and the 12‐population data set (data set 2) was used to test trait correlations and model trait distributions.

Abbreviations: $\frac{1}{\text{AI}},\;\frac{1}{\text{aridity}\;\text{index}}$; *P*_DM_, precipitation of the driest month; *P*_MA_, mean annual precipitation; *T*_MAX_, maximum temperature of the warmest month.

### 2.3 Trait measurements

Traits were measured in March 2017 on *C. calophylla* trees that were 29 months old and 2–3 m tall. For each individual tree, we removed a north facing, mid‐canopy side branch at its intersection with the main stem. The side branch was removed in the morning (between 8 a.m. and 12 noon), stored in a cool box, and measured in the afternoon (between 12 noon and 6 p.m.). For each side branch, we collected data for seven separate traits: leaf‐level spectrometer readings to calculate two indices (photochemical reflectance index (PRI) and normalized difference vegetation index (NDVI)), leaf size (LS), specific leaf area (SLA), integrated water‐use efficiency (δ^13^C), nitrogen concentration (N_CONC_), and wood density (WD). All seven traits have shown close association to climate in past studies. Photochemical reflectance index (PRI) is a spectral physiological index that is an indicator of vegetation stress based on its sensitivity to radiance‐use efficiency (RUE) and the xanthophyll cycle (Gamon, Serrano, & Surfus, 1997; Garbulsky, Peñuelas, Gamon, Inoue, & Filella, 2011). Leaf‐level normalized difference vegetation index (NDVI), which is generally used to measure chlorophyll content by quantifying leaf greenness, and is closely related to fraction of absorbed photosynthetically active radiation (FPAR) (Myneni et al., 2002; Peng & Gitelson, 2012). While not technically functional traits (PRI and NDVI), traits based on spectral properties of leaves can be indicative of photosynthetic activity and plant stress, and from hereon, we include these complex traits as functional traits for ease of discussion. High water‐use efficiency (WUE) is the link between photosynthesis and evaporation (Yang et al., 2016) that translates to climatic tolerance under water limitation. Leaf size may be beneficial in certain circumstances as it can act as a major determinant of boundary layer thickness, particularly in low‐wind conditions. Larger leaves generally experience higher temperatures, increasing carboxylation and other catabolic processes such as dark respiration (Jordan & Smith, 1995; Jones, 2013). Specific leaf area (SLA) varies across global climate gradients (Wright et al., 2004), and high SLA values increase tree susceptibility to drought‐induced mortality (Greenwood et al., 2017). Water‐use efficiency is correlated with δ^13^C (an isotopic signature measuring the ratio of ^13^C and ^12^C (Farquhar & Richards, 1984)) and relates to leaf gas exchange properties (Cernusak et al., 2013; Diefendorf, Mueller, Wing, Koch, & Freeman, 2010). Nitrogen concentration (N_CONC_) is indicative of growth. Leaf nitrogen plays an important role in leaf physiological processes such as photosynthesis, respiration, and transpiration (Wang et al., 2016) and is an indicator of productivity (Ramoelo, Skidmore, Schlerf, Mathieu, & Heitkönig, 2011). The association between wood density (WD) and drought is more complicated and context dependent, because it is associated with many ecological signals (Brodersen, 2016; Gleason et al., 2016). Generally, high WD is associated with lower susceptibility to drought (Greenwood et al., 2017; Hacke, Sperry, Pockman, Davis, & McCulloh, 2001), resulting in higher fitness in hot–dry environments compared to trees with low WD by decreasing embolism rates (Cochard, Casella, & Mencuccini, 2007).

A field spectroradiometer (ASD standard‐resolution FieldSpec4, Malvern Panalytical) was used to measure leaf reflectance in the visible and reflected infrared spectral regions with 2,151 narrow bands (10 nm full width at half maximum) and 1 nm spacing between band centers. Measurements were made for three leaves using a leaf‐clip attachment with its own light source and calibrated to % reflectance using data collected from a Spectralon white reference panel. Means for all bands among the three leaves were calculated for each individual tree. Specific wavelengths were used to estimate the PRI and modified red‐edge NDVI. The PRI was calculated with the following equation; *R*_xxx_ is the % reflectance at xxx nm (Gamon, Penuelas, & Field, 1992; Gamon et al., 1997):

$$\text{PRI}\;=\frac{R_{531}-R_{570}}{R_{531}+R_{570}}$$

The modified red‐edge NDVI was calculated using the following equation (Sims & Gamon, 2002):

$${\text{mND}}_{705}=\frac{R_{750}-R_{705}}{R_{750}+R_{705}-2\times R_{445}}$$

and was developed as an improvement to the standard NDVI to provide a more robust estimate of chlorophyll content (Tucker, 1979) across a wide range of species and leaf structures (Sims & Gamon, 2002). Henceforth, this index will be referred to as “NDVI” in the text.

Specific leaf area (SLA) was measured on three fully matured leaves that were representative of the branch. After removing half of the petiole with a razor, the leaves were scanned into a computer using a Canon flatbed scanner (model # LiDE220) at 50 dpi. The leaves were kept in an airtight box with silica gel until they could be dried in an oven at 70°C for 48 hr. δ^13^C and nitrogen concentration (N_CONC_) were measured from leaves dried using a benchtop freeze dryer (Alpha 1‐4 LDplus Laboratory Freeze Dryer, Martin Christ). The leaves were grounded into a fine powder using a cyclotec mill (Foss Analytics) and sent for isotope analysis (ANU Isotope Laboratory) using a coupled EA‐MS system (EA 1110 Carlo Erba; Micromass Isochrom). For wood density, a 3–4 cm piece of the thickest part of the branch was removed, excluding areas that included knots, the bark was removed, and the volume measured using the water displacement method. The piece of wood was then dried in a 70°C oven for 7 days before measuring for dry weight. Final wood density was calculated by dividing the dry weight by the volume (g/cm^3^).

### 2.4 Statistical analyses

Using data set 1 with four populations, mixed‐effects linear models were applied to estimate differences between populations using the *lme* function in the *nlme* package in R (R Core Team, 2018). For all linear models, family was considered a random effect and population considered a fixed effect. Post hoc Tukey tests were performed on the mixed‐effects model results using the *glht* function in the *multcomp* package in R to confirm differences among populations.

We estimated the family level narrow‐sense heritability and the relative effect of selection on each of the seven traits. Narrow‐sense heritability (${\hat{h}}^{2}$) captures the proportion of genetic variation attributed to additive genetic variance (Lynch & Walsh, 1998). Here, we used data set 1 with four populations within ASReml version 4.1 (Gilmour & Dutkowski, 2004). Initial assessment of model fit was conducted using the following univariate random model:

$$y_{\mathrm{ijkl}}=\mu +b_{i}+p_{j}+f_{i.k}+b_{i}\times f_{i.k}+e_{\mathrm{ijk}},$$

where $b_{i}$ is the random effect of the ith block, $p_{j}$ is the random effect of the jth population, $f_{i.k}$ is the random effect of the kth family within the ith population, $b_{i}\times f_{i.k}$ is the block × family interaction effect, and $e_{\mathrm{ijk}}$ is the random error. Narrow‐sense heritability was estimated using the following equation:

$${\hat{h}}^{2}=\frac{2.5\times \sigma_{\text{fam}}^{2}}{\sigma_{\text{fam}}^{2}+\sigma_{\text{fam}\times \text{block}}^{2}+\sigma_{\text{error}}^{2}}$$

where ${\hat{h}}^{2}$ is the narrow‐sense heritability, $\sigma_{\text{fam}}^{2}$ is the family within population variance component, $\sigma_{\text{fam}\times \text{block}}^{2}$ is the family × block interaction, and $\sigma_{\text{error}}^{2}$ is the error component of variance. Eucalypts are known to have a mixed mating system; therefore, a coefficient of relationship (*ρ* = 1/2.5) was assumed to correct for selfing effects of about 30%, which, if not corrected for, could result in inflated estimates of heritability for growth traits (Bush, Kain, Matheson, & Kanowski, 2011; Costa e Silva, Hardner, & Potts, 2010; Griffin & Cotterill, 1988; Hodge, Volker, Potts, & Owen, 1996). Significance of family variance components was determined using a log‐likelihood ratio test as described in the ASReml manual by dropping the family component from the model and comparing these log‐likelihood results to the full model.

Using data set 1 with four populations, we developed two principal components analyses (PCA) based on family means to understand the relationship between populations, traits, and climate and used to inform the modeling step described below. The first PCA was performed with the seven measured traits (LS, WD, δ^13^C, N_CONC_, SLA, PRI, and NDVI) to view relative explanatory power of population differentiation using traits alone and identify possible trait–trait relationships. A second PCA explored the relationship between climate and traits. First, climate data were downloaded from WorldClim (Fick & Hijmans, 2017) (precipitation of the driest month (*P*_DM_), mean annual precipitation (*P*_MA_), precipitation variation (*P*_RANGE_), mean annual temperature (*T*_MA_), maximum temperature of the warmest month (*T*_MAX_), and temperature variation (*T*_RANGE_)), and Consortium for Spatial Information (CGIAR‐CSI) (aridity index (AI); transformed to $\frac{1}{\mathrm{AI}}$), and the data were extracted for the location of each source population. For this study, AI was defined as $\frac{\text{mean}\;\text{annual}\;\text{precipitation}}{\text{mean}\;\text{annual}\;\text{evapotranspiration}}$ and we used the inverse of this ratio because it is more intuitive to interpret (i.e., the higher the number the more arid the climate). Four environmental variables (*P*_DM_, *P*_MA_, *T*_MAX_, $\frac{1}{\text{AI}}$) were included in the second PCA that was performed to characterize underlying patterns of correlation among independent and dependant variables and to understand the correlation between climate and traits. We used the *prcomp* function in R and plotted the PCA using the *ggbiplot* function.

Using data set 2 with 12 populations, we explored four sets of correlations among seven traits. We deliberately use the term “correlation” to describe the relationship between three of the trait pairs because coordination implies established mechanistic relationship, like those in the LES and have not been explicitly established for three of the correlations explored in detail here. The first correlation was between δ^13^C and PRI. PRI has been shown to be a measure of water stress and indirectly photosynthetic radiation‐use efficiency, while δ^13^C is a well‐known measure of water‐use efficiency. Together, radiation‐use efficiency and water‐use efficiency have been shown to be tightly correlated in wheat and soybean (Caviglia, Sadras, & Andrade, 2004). The second was LS and NDVI, which have similar relationships with fast‐growth syndrome. Plants that have a fast‐growth strategy also grow larger leaves (Cornelissen, 1999; Wright et al., 2017), as light is by far the most limiting resource for tree growth (Pacala et al. 1996), and light capture depends on the size of the leaves (Falster & Westoby, 2003; Pearcy, Muraoka, & Valladares, 2005; Pearcy, Valladares, Wright, & De Paulis, 2005). We expect a similar relationship for NDVI and have been shown to be a good predictor of aboveground biomass in trees (Goetz & Prince, 1996; Malstrom et al., 1997; Wang, Rich, Price, & Kettle, 2004). Therefore, we would expect NDVI and LS to be highly correlated. The third was WD and N_CONC_, where we expect that the plants with lower WD would also have higher N_CONC_ (e.g., (Beets, Gilchrist, & Jeffreys, 2001; Lindstrom, 1996)), where faster wood growth can be attributed to higher N_CONC_. The fourth association tested was between SLA and N_CONC_, which have been well described within the LES paradigm (Reich, 2014; Reich et al., 1997). Trait correlations were tested using linear models (function *lm*) on family means between the traits in R. The outputs were plotted using base R commands.

To estimate and predict trait change through geographic space and time, general additive models (GAM) were used to detect nonlinear relationships between trait and climate. A GAM uses a robust and efficient smoothing parameter (Wood, Pya, & Säfken, 2016), and we were able to fit nonlinear smoothing terms using regression splines without any a priori assumptions. This property makes GAMs very useful for detecting nonlinear responses across a species distribution, and trait variation is generally nonlinearly associated with environment (Moran, Hartig, & Bell, 2016). The GAMs were performed using data set 2 with 12 populations and current bioclim variables downloaded from WorldClim (*P*_DM_, *P*_MA_, *P*_RANGE_, *T*_MA_, *T*_MAX_, *T*_RANGE_) and CGIAR‐CSI ($\frac{1}{\text{AI}}$) at the sampled population sources. Separate GAMs were developed for each trait individually, in order to evaluate how each trait might respond to climate change. We used the *GAM* function in the MGCV v1.8‐24 package in R (Wood, 2011) to perform the analyses. To minimize overfitting of the data, we only explored different combinations of up to three environmental variables and bound degree of smoothness to three for each variable (Araujo, Pearson, Thuiller, & Erhard, 2005), using cubic regression splines to control the degree of smoothness (Wood, 2006). The model that gave the highest *R* ^2^ and deviance explained are reported and discussed. Extrapolation of the GAM model outputs was performed using the *predict* function in R on the climate rasters of the distribution of *C. calophylla*, providing a predicted trait value for each pixel under current conditions. These outputs were visualized using ggplot2 (Wickham, 2016).

To predict the effects of climate change on trait distributions, raster files from 2070 under emissions scenario RCP 8.5 were downloaded from WorldClim under the CCSM4 simulation. A future aridity index raster file was created using the ENVIREM package in R. The outputs of the GAM models were used to predict future trait distributions by substituting the current rasters with the future climate predicted rasters from RCP 8.5 CCSM4 simulation. In order to visualize where trait changes are predicted to occur, the difference between current and future trait distribution was estimated by subtracting the current distribution from the future distribution and dividing by the current trait values. This provides a proportional change value for each pixel in the distribution. These maps indicate the direction (e.g., higher or lower SLA) of trait evolution needed in order to maintain its predicted genotypic relationship with climate into the future. These outputs were standardized (proportional change), so the direction and proportional change among traits can be compared.

In order to test the hypothesis of possible uncoupling of trait correlation in the future, we compared the relationships between pairs of traits under the GAM‐derived future trait values and GAM‐derived current trait values for all pixels within the distribution (*n* = 106,833). The function *linearHypothesis* in the *car* package was used to test whether the slopes of the trait correlations were different between time frames. The similarity of the slopes between the current spatial patterns of the GAM‐derived traits and the traits measured on the 12 populations in the plantation was also assessed.

## 3 RESULTS

### 3.1 Trait differentiation and heritability

**Figure 2:**
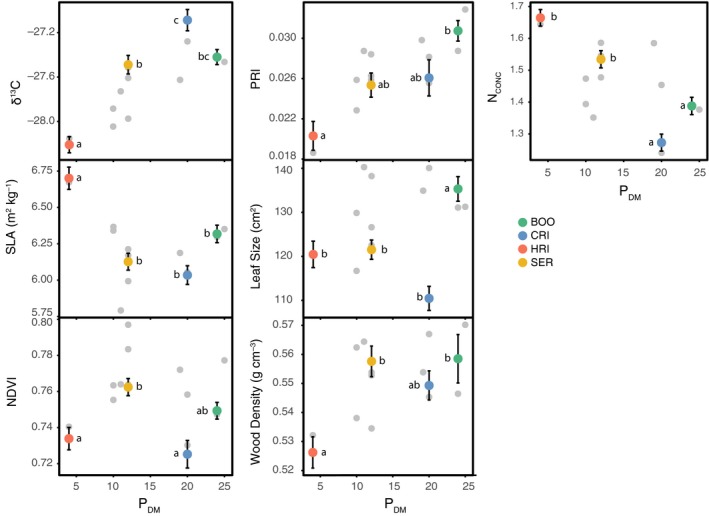
Trait means for 12 populations distributed along a gradient of *P*_DM_ from the population's climate‐of‐origin. Data set 1 has colored symbols for the four populations (BOO = cool–wet; CRI = cool–dry; HRI = warm–dry; SER = warm–wet) with SE of the mean based on 10 families with 12 replicates. Data set 2 is shown with gray dots for the means of 12 populations. Letters indicate significant differences between populations from a post hoc Tukey's test (*a* = 0.05) on a mixed‐effects linear model with family as the random variable. N_CONC_, concentration of nitrogen (%); NDVI, normalized difference vegetation index; *P*_DM_, precipitation of the driest month (mm); PRI, photochemical reflectance index; SLA, specific leaf area; WD, wood density; δ^13^C, ratio of ^13^C versus ^12^C

All seven traits showed patterns of population differentiation (Figure 2). The two most extreme populations in relation to precipitation of the driest month (*P*_DM_) (populations from HRI and BOO) were significantly different for all traits except for NDVI. Three traits (δ^13^C, PRI, and N_CONC_) showed a significant linear response with at least one climate variable (Table S1).

Narrow‐sense heritability ranged between 0.08 and 0.21 for the seven traits. While heritability for SLA (${\hat{h}}^{2}$ = 0.08 ± 0.08 SE; *p* = NA) and PRI (0.11 ± 0.08; *p* = .07) was not significant under the log‐likelihood ratio test, the other five traits showed heritable patterns that were significant. The estimates of heritability were on a continuum with N_CONC_ (0.22 ± 0.09; *p* = .001), LS (0.18 ± 0.1; *p* = .007), and δ^13^C (0.17 ± 0.08; *p* = .003) the greatest, NDVI (0.15 ± 0.08; *p* = .007) and WD (0.12 ± 0.08; *p* = .02) intermediate, and SLA and PRI the lowest.

### 3.2 Trait correlation

**Figure 3:**
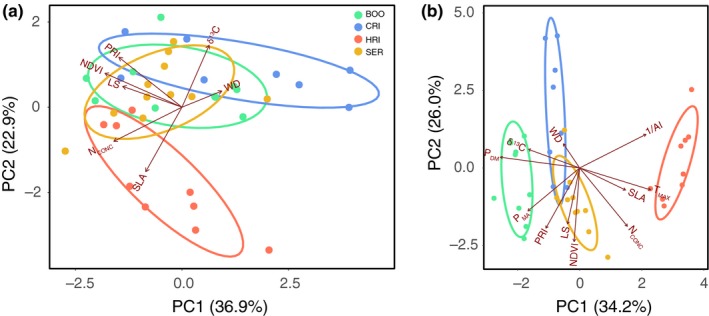
Principal components analysis (PCA) of family means for four populations with seven traits (a), and seven traits and four climate variables (b). Each point represents the mean for each family estimated from 10 to 12 individuals. Families are colored by population, and the ellipse encapsulates 95% of the population variation. Population and trait abbreviations are defined in Table 1 and Figure 2, respectively

The principal components analysis shows clustering of families within populations and associations between traits. The first two axes of the PCA explained 59.8% of the variation among the seven functional traits (Figure 3a). The PCA shows partially overlapping population clusters that are separating along the PC2 axis, explaining 22.9% of the variation with the three traits SLA, δ^13^C, and N_CONC_ being the most likely traits differentiating the populations. The HRI (warm, dry climate) population was clearly differentiated from the cool, dry climate (CRI) population, while BOO and SER were intermediate. Along the PC1 axis, the traits NDVI (axis 1 loadings = −0.52), N_CONC_ (−0.46), PRI (−0.42), and LS (−0.40) displayed a greater loading than the others, indicating that these traits were found to be more indicative of within population differentiation. Along the PC2 axis, the traits SLA (axis 2 loadings = −0.56), δ^13^C (0.53), and PRI (0.43) displayed the greatest loadings, indicative of stronger among populations differentiation. The WD trait has the third lowest loading (0.27) on the PC1 axis and the lowest loading (0.14) on the PC2 axis.

**Figure 4:**
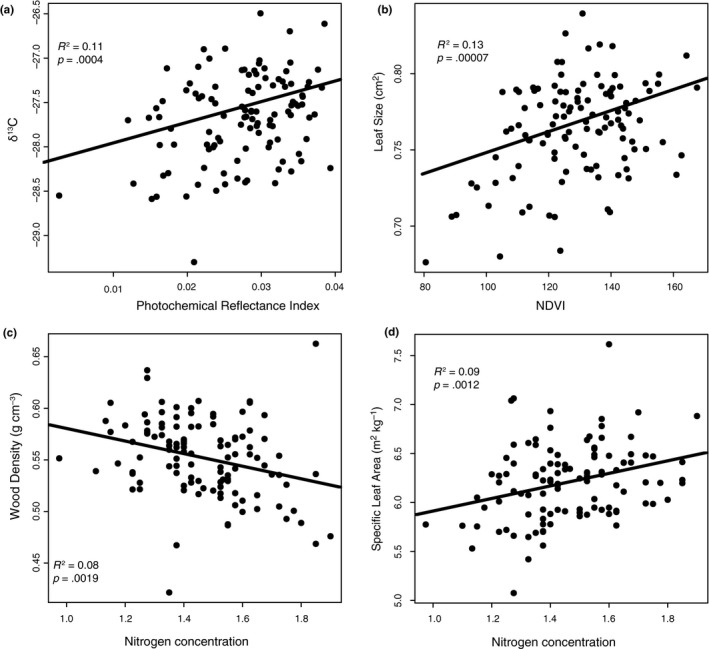
Correlation between traits among 114 families from 12 populations. Each point represents a family mean value from four trees. The line of best fit, *R* ^2^, and *p*‐value are calculated from a linear model

All three trait correlations and the trait coordination were significantly correlated (Figure 4). There was weak but significant correlation between δ^13^C and PRI (Figure 4a), LS and NDVI (Figure 4b), WD and N_CONC_ (Figure 4c), and SLA and N_CONC_ (Figure 4d). Three of the correlations showed a positive relationship between the two traits. The only relationship that showed a negative correlation was between the WD and N_CONC_ traits.

### 3.3 Adaptation to climate

When the PCA included trait and climate variables, the separation of populations became more apparent (Figure 3b). The warm, dry climate (HRI) population was the most divergent from the other three populations. The PC1 best differentiates the populations with 32.4% of the variance explained by a combination of trait and climate variables. We find that SLA is correlated with *T*_MAX_, δ^13^C is correlated with *P*_DM_, PRI is correlated with *P*_MA_, and no trait is correlated with $\frac{1}{\text{AI}}$. *P*_DM_ had the greatest loading for PC1 closely followed by *T*_MAX_ and $\frac{1}{\text{AI}}$ (*P*_DM_ = −0.48, *T*_MAX_ = 0.43, $\frac{1}{\text{AI}}$ = 0.40) then *P*_MA_ (loading = −0.31), which had similar loading to δ^13^C (loading = −0.31) and N_CONC_ (loading = −0.29) traits. NDVI was the variable with the greatest loading on the PC2 (loading = −0.51) followed by PRI (loading = −0.42), and these variables show more variation within populations than among populations, as exhibited by the long population ellipses that follow the PC2 axis.

**Figure 5:**
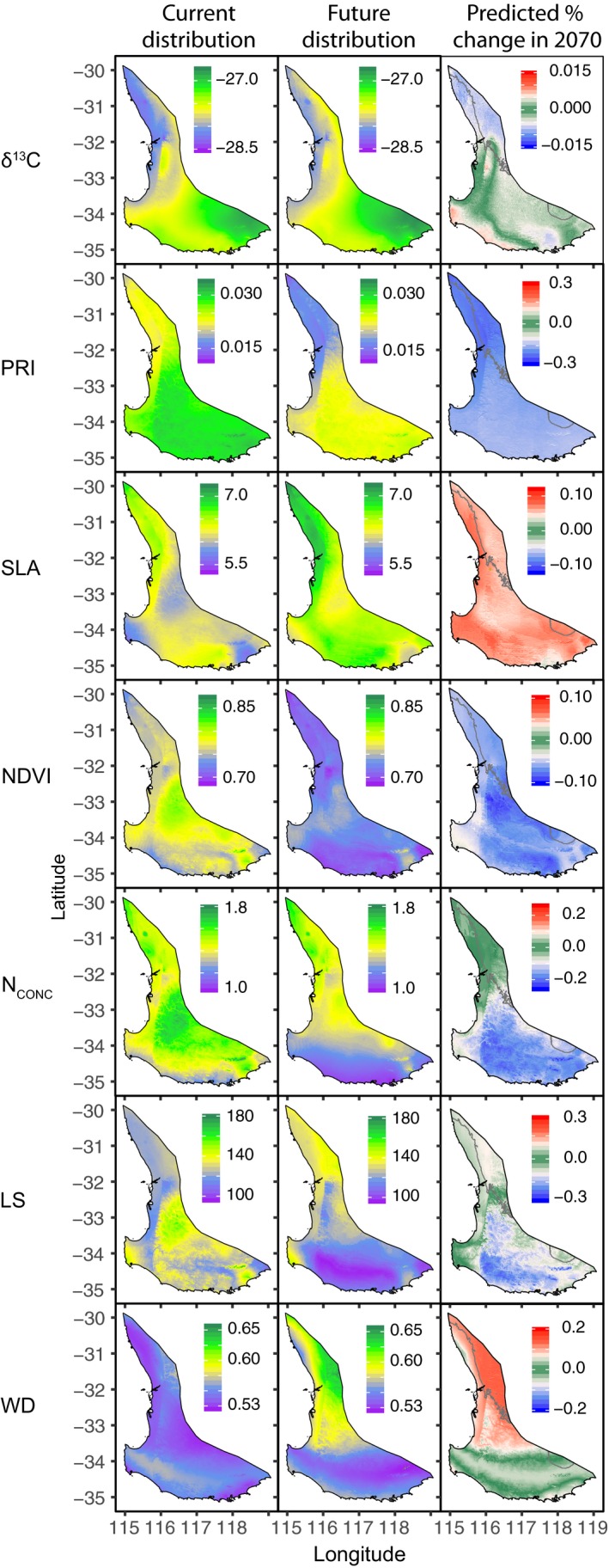
Current, future, and predicted % change in 2070 trait values for the seven traits across *Corymbia calophylla*'s distribution using GAM and the relative change of those traits predicted from current to future climate. Gray lines denote climate space that exceeds current species distribution limits. Population and trait abbreviations are defined in Table 1 and Figure 2, respectively

The GAM analysis revealed different patterns of trait change through the landscape among all traits (Figure 5), and different trait responses to individual climate variables (Figure S1). At least one temperature and one precipitation variable were presented for all traits except for δ^13^C (only precipitation variables) and PRI (only temperature variables). The most common climate variable incorporated into the final GAM for each trait was *P*_MA_, and the least common was $\frac{1}{\text{AI}}$ (Table 2). The deviance explained by the GAM analysis was high (*R* ^2^ > 0.6) for all traits. The GAM analysis explained greater than ca. 85% of the deviance for SLA, δ^13^C, N, NDVI, and LS.

**Table 2:** Variable significance and model performance for each general additive model (GAM) among seven traits

Trait	*T*_MA_	*T*_MAX_	*T*_RANGE_	*P*_MA_	*P*_DM_	*P*_RANGE_	$\frac{1}{\text{AI}}$	*R* ^2^	dev
δ^13^C	–	–	–	9.542*	2.719	5.472*	–	0.929	96.5
PRI	–	15.466**	8.191*	–	–	–	–	0.604	67.6
NDVI	14.404**	–	–	7.123*	15.346**	–	–	0.874	94.8
LS	12.178*	–	7.016*	–	5.234	–	–	0.846	94.5
WD	–	7.2*	–	9.408*	–	–	–	0.682	79.5
N_CONC_	17.087**	–	8.138*	12.264**	–	–	–	0.896	94.6
SLA	–	19.64**	14.49*	16.37**	–	–	–	0.951	97.9

Note

*F*‐values are provided for each climate variable used in the model with their significance level (*<0.05; **<0.01).

Abbreviations: dev, deviance explained; LS, leaf size; N_CONC_, nitrogen concentration (%); NDVI, normalized difference vegetation index; PRI, photochemical reflectance index; SLA, specific leaf area; WD, wood density; δ^13^C, ratio of ^13^C versus ^12^C.

The climate factors are as follows: *T*_MA_, mean annual temperature; *T*_MAX_, maximum temperature of the warmest month; *T*_RANGE_, temperature variation; *P*_MA_, mean annual precipitation; *P*_DM_, precipitation of the driest month; *P*_RANGE_, precipitation variation

All traits showed altered distributions between current and future climates (Figure 5). The traits PRI, NDVI, and N_CONC_ mostly had a reduction in their trait values throughout the distribution, while SLA was the only trait that consistently increased in the future. The δ^13^C, LA, and WD traits had reduced trait values in some geographic regions but increased values in other regions. The magnitude of trait change was greatest for PRI, LS, and N_CONC_, which are predicted to change by 25%–30%. In contrast, δ^13^C and NDVI had the lowest magnitude of predicted trait change with 1.5% and 10% change, respectively. There are portions of the range that are predicted to be outside of current climate conditions (shown with a gray line in Figure 5); therefore, our predictions in these regions should be treated with caution.

**Figure 6:**
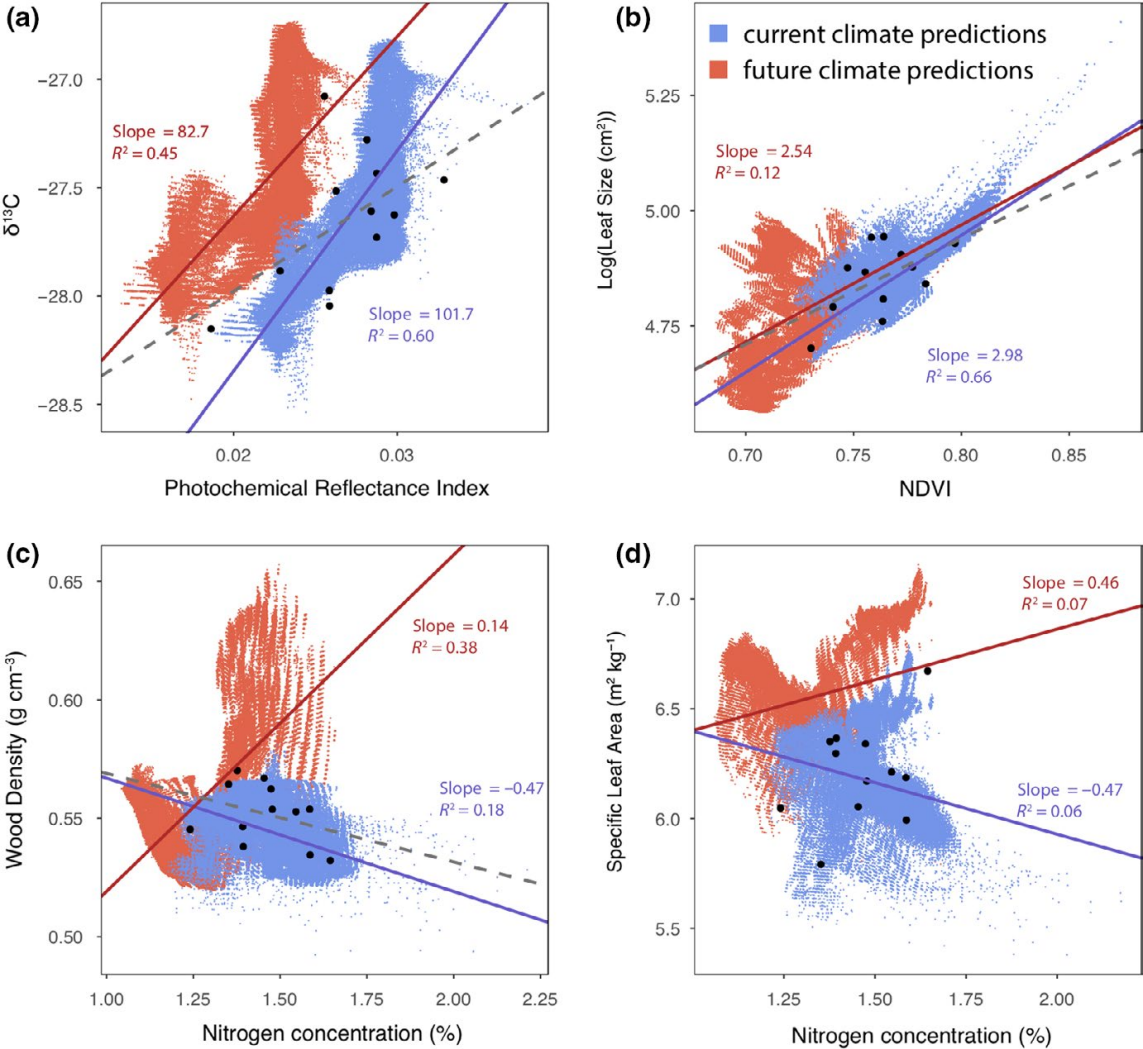
Predicted trait values across the spatial distribution of *Corymbia calophylla* using current climate data (blue) and future climate data from 2070 (red) estimated by GAM analysis. Each blue and red dot represents a single pixel from the current and future maps, respectively, in Figure 5. Black dots are population‐level trait values. Only significant (*p* < .05) lines of best fit are shown for each of the three data sets

All four of the tested trait correlations were highly significant when evaluating the spatial relationships (both current and future) between predicted trait distributions (Figure 6; *p* = 2.2e^−16^). However, the SLA/N_CONC_ slopes explained only 6%–7% of the variation, indicating high levels of variability among the data. In contrast, the δ^13^C/PRI slopes explained 45%–60% of the variation for future and current predictions, respectively (Figure 6). The spatially predicted slopes of the trait correlations compared to the sampled populations only differed for the δ^13^C/PRI correlation (Figure 6a). Trait correlations are predicted to differ between current and future conditions for all trait pairs except LS/NDVI (Figure 6b). These differences are not uniform across correlations, as the WD/N_CONC_ slope changes from a negative slope to a positive slope, and the δ^13^C/PRI slope appears to shift along the PRI axis. The traits associated with the LES also change from a negative to a positive slope, but this might be attributable to the large amount of variation in SLA.

## 4 DISCUSSION

We found evidence to support adaptation of functional traits in *C. calophylla* across populations in southwest Western Australia. These patterns of adaptation are consistent with previous studies of genetics, growth, and physiology of *C. calophylla* (Ahrens, Byrne, et al., 2019; Ahrens, Mazanec, et al., 2019; Aspinwall et al., 2017; Blackman et al., 2017). This study builds upon previous research by focusing on the genetic determination of functional traits, elucidating the relationship between functional traits, determining the relationship between functional traits and climate, and predicting genetically determined trait distributions under current and future climates. By evaluating trait variation in a common experimental site, we were able to evaluate trait heritability and genotypic differences among populations, with minimal interference from environmental factors. Therefore, the trait values measured are accurate for the climate‐of‐plantation and are representative of genotypic signatures of local adaptation. There were significant differences among populations for all traits, and all traits exhibited associations with climate‐of‐origin but some traits were related to climate‐of‐origin in unexpected ways. We found five of the seven traits had significant heritability indicating an ability to respond to selection and adapt to climate. However, traits differed in their heritability and trajectory along climate space. We predict trait coordination and trait correlation decoupling in the future, where traits may change at different rates to adjust to future climates.

### 4.1 Trait differentiation and heritability

Three of the traits measured (NDVI, PRI, and LS) exhibit tendencies that follow with previous studies exploring the adaptation of functional traits adapted to hot, dry environments. For example, the lower NDVI from a hot, dry climate compared to a hot, wet climate suggests an adaptive strategy among populations. This finding agrees with Ahrens, Mazanec, et al. (2019), which shows a strong pattern of slow‐growth strategies among northern populations, which is characteristic of hot and arid environments (King, 1990; Moles, 2018; Reich, 2014). The population differences found in PRI are best explained by T_MAX_ (see the GAM analysis) and clearly show a lower value in the population from a hot, dry climate (HRI), indicating a lower photosynthetic rate due to reduced radiation‐use efficiency (Garbulsky et al., 2011). This is in agreement with Aspinwall et al. (2017), who found higher photosynthetic rates in the cool, wet population compared to the hot, dry population. Lastly, LS has received significant attention recently, as worldwide LS patterns show significant relationships with temperature and latitude (Wright et al., 2017). In *C. calophylla*, LS is correlated with temperature of origin and is similar to *Eucalyptus loxophleba* where LS decreased with temperature (Steane et al., 2017). Greater leaf size may confer advantages with greater gas exchange (Zwieniecki, 2004) and protection against herbivory (Moles & Westoby, 2000), while smaller leaf size could lead to overheating avoidance (Okajima, Taneda, Noguchi, & Terashima, 2012), and leaf size is known to be coordinated with essential plant architecture such as canopy size and plant hydraulics (Jensen & Zwieniecki, 2013; Sack et al., 2012). However, the contrast with the broad findings in Wright et al. (2017) was not surprising because the broader, across species patterns for complex variable traits may not be present within species (Moles, 2018). In fact, many studies exploring how traits change with environment have been performed on unrelated species (e.g., Atkin et al., 2015; Díaz et al., 2016; Wright et al., 2017), while intraspecific trait variation is “largely ignored” in trait‐based plant ecology (Shipley et al., 2016), providing limited information on evolutionary responses to climate (Moran et al., 2016).

Some functional traits showed a different pattern in relationship to climate‐of‐origin than expected. Four traits (δ^13^C, SLA, N_CONC_, WD) showed values for the hot, dry climate (HRI) indicative of higher rainfall areas. However, this is likely due to populations of *C. calophylla* from the hot, dry climate (HRI) having different adaptive mechanisms than anticipated. Carbon isotope composition (δ^13^C) is tightly correlated with WUE, particularly in field studies (Rundel & Sharifi, 1993), and our findings suggest that HRI has the lowest WUE, when we expected it to have the highest WUE. Likewise, the LES traits (SLA and N_CONC_) within the HRI population were different than expected; the high values for SLA and N_CONC_ measurements are indicative of a population that is more susceptible to drought conditions (Greenwood et al., 2017). While we do find differentiation between populations for SLA, this differentiation does not seem to be heritable and confirms findings that SLA is highly plastic (Shipley, 2000). In addition, WD exhibited the opposite pattern than was expected, as the wood was less dense in the population from the hotter, drier climate. We expected northern *C. calophylla* populations that occur in hotter, drier conditions to exhibit denser wood to increase cavitation resistance (Hacke et al., 2001) and increase survival in harsh climates (Cornwell & Ackerly, 2009; Hacke et al., 2001). Even though we found differences between populations for the WD trait with some evidence of heritability, the WD differences (c. 0.40–0.65) are small and may result in similar *P*_50_ estimates (c. −2 to −2.5; *P*_50_ is the point at which plants lose 50% of their conductance (Hacke et al., 2001)), suggesting that the biological difference between the WD measurements may be negligible. Overall, these patterns were unexpected and our finding that the HRI population from the hotter, drier climate was at the end of the trait spectrum where we expected cool, wet populations may be attributed to one or several possible explanations: (a) patterns of isolation might have affected current trait distributions, and genetic drift might overcome selection in some small, isolated populations (Lanfear, Kokko, & Eyre‐Walker, 2014); (b) other traits may be involved in the adaptation to hotter, drier climates for this species; (c) these traits might be plastic; or (d) a whole‐plant leaf‐area process in which the northern populations grow structurally inexpensive (thin) leaves with low leaf longevity provides ways in which populations can adapt to hot, dry climates, as found in other trees (Wolfe, Sperry, & Kursar, 2016).

All but two traits (SLA and PRI) had a narrow‐sense heritability greater than zero, indicating that evolutionary change can occur through processes of natural selection. In particular, the heritability of WUE, as indicated by δ^13^C, is an important finding that has ramifications for the species as the climate changes, particularly in a Mediterranean‐type climate, where rainfall is seasonal and climate shifts are predicted (Klausmeyer & Shaw, 2009). However, heritability was not found to be consistent among traits, indicating that our hypothesis of different levels of variability among traits is accepted. In general, these heritabilities are similar to the heritability of height (0.14 ± 0.03 SE), diameter (0.12 ± 0.03), and blight resistance (0.08 ± 0.03) based on a greater sampling effort (24 seedlings per family; 3,960 trees) at the same experimental plantation (Ahrens, Mazanec, et al., 2019). Overall, the heritability continuum measured here suggests that selection pressure due to climate will affect each trait differently, leading to novel patterns of local adaptation and trait combinations.

The presence of variation in trait means and heritability describes a system in which the species is able to adapt to a future climate. However, no two traits shared the same explanatory climate variables or modeled associations, resulting in different predicted distributions. We found that some traits would need to evolve at a greater rate than others in order to maintain current trait–climate associations under future conditions (see the change factor in Figure 5). Some traits may lag behind the climate change front, while others may be able to adapt to future climates, depending on the level of individual trait heritability, strength of climate selection pressure on the trait, and the amount of standing genetic variation present within the genes that control the trait. For example, the SLA trait would need to change as much as 12% to keep up with the changing climate, but SLA is effectively not heritable, so the change needed is unlikely to happen via selection pressure. On the other hand, N_CONC_ would need to change its trait value by −25% and has one of the higher estimates of heritability in the study, indicating that this trait might track with environment if the selection pressure is strong. Overall, our observations are consistent with the concept that some traits will be more limiting than others in relation to a species’ adaptive capacity to respond to climate change.

### 4.2 Trait correlation

Our estimates of trait correlations are consistent with other studies and are in‐line with our expectations. However, the traits measured differentially respond to climate and our predictions that climate change may affect each trait independently were supported. Our data suggest that traits will need to adapt to new climates at different rates and in different patterns. This is a concern for traits that are known to be dependent on one another, such as those in the LES. However, the other correlations may be able to independently adapt to new conditions. This is particularly concerning for traits that are mechanistically dependent on one another because the coordinated traits will either need to decouple or be limited by one another. We were able to establish the presence of a relationship between PRI (RUE) and δ^13^C (WUE). This correlation is indicative of populations having different photosynthetic inhibition under different light conditions (Grace et al., 2007). In the future, we anticipate that this correlation between PRI and δ^13^C will decouple, as a greater shift in PRI is predicted compared to δ^13^C, but this correlation may not change under new climate conditions because of the effective nonheritability of PRI and the very small change for δ^13^C.

Current patterns of coordination between SLA and N_CONC_ are consistent with the overall patterns within the LES paradigm (Reich, 2014; Reich et al., 1997), in that high N is associated with high SLA however the pattern's association with climate is the opposite as expected. The combination of SLA and N_mass_ has been shown to be a good predictor of net photosynthesis (A_max_) on a per mass basis (Reich & Walters, 1994; Reich et al., 1997), and lower SLA is due to many anatomical features (e.g., larger cell sizes, greater major vein allocation, greater numbers of mesophyll cell layers, and higher cell mass densities (John et al., 2017)), although we found that SLA is not heritable and highly variable. Our findings suggest that the adaptive potential of these two LES traits could be limited by one another, or SLA (not heritable) could dynamically match *N* (heritable) concentrations through plasticity.

We also expected to find significant correlation between LS and NDVI. As such, we revealed the BOO population (cool–wet climate‐of‐origin and the fastest growing population; Ahrens, Mazanec, et al., 2019) as having larger leaves and higher NDVI, which is indicative of higher biomass. This is the only trait correlation that we predict to remain intact in the future.

The pattern between WD and N_CONC_ was also as expected, in that wood density decreased with increasing N_CONC_. However, the predicted correlation between WD and N_CONC_ shows a nearly perpendicular change, indicating that the two traits will be required to evolve in opposite directions. This confirms that WD is a difficult trait to predict due to its association with many ecological signals (Brodersen, 2016; Gleason et al., 2016) and that other mechanisms, aside from climate, are likely important for selection of the WD trait.

All of the current trait correlations were highly significant but with low explanatory power, indicating that the variation between correlated traits is high, and that the correlation has some leeway for traits to change without affecting patterns of adaptation. Overall, predicted trait correlations exhibit contrasting prediction scenarios, which could force some traits to change disproportionately compared to their counterparts. These findings suggest that if these trait correlations are dependent on one another that they might be a hindrance to adapting to novel climate conditions. On the other hand, if the correlated traits can evolve independently, the different trait heritability levels suggest that some traits are more genetically determined than others, resulting in different trait combinations within populations than what has been measured.

## 5 CONCLUSION

Understanding mechanistic patterns of plant traits that undergo processes of natural selection can broadly enhance our understanding of species distributional predictions to inform maintenance of forest ecosystem function under future climate scenarios. Our results suggest that functional traits have contrasting genotypic patterns and will be subjected to different climate selection pressures, which may negatively affect current forest structure and function due to lower working optimum for functional traits. Even though we were able to identify significant adaptive variation and differential trait responses correlated with patterns of precipitation and temperature, demonstrating adaptive capacity to climate change, we reveal that traits are independently associated with different climate factors. Therefore, some trait correlations and their idiosyncratic relationships may be disrupted under future climate scenarios, suggesting that genetic constraints, selection pressure, and trait correlation limitations will affect trait evolution and patterns of adaptation in the future.

## CONFLICT OF INTEREST

None declared.

## AUTHOR CONTRIBUTIONS

CA and PR developed the idea. All authors collected the data. CA, RM, and MA analyzed the data. CA wrote the first draft, and all authors edited and intellectually contributed to the final manuscript.

## Data Availability

Data for this study are available at https://doi.org/10.5061/dryad.nk98sf7pv and include two files, one to calculate heritabilities with four populations and one for the modeling component with twelve populations. Metadata are included for all populations within each file including environmental factors and GPS locales.
