# Supplementary material for: Plant functional traits differ in adaptability and are predicted to be differentially affected by climate change
Source: Ecol Evol. 2019 Nov 28;10(1):232–48. doi: 10.1002/ece3.5890 (PMC6972804; doi:10.1002/ece3.5890)
Supplement: Supplementary file 1 [file ECE3-10-232-s001.docx]

Journal of Ecology

Supplementary information.

Plant functional traits differ in adaptability and are predicted to be differentially affected by climate change

Ahrens et al.

Table S1. F-values for linear relationships between plant traits and environment. Significance are indicated by * < 0.05 and ** < 0.01.

| Trait | TMAX | PDM | PMA | 1/AI | TMAX*PDM |
| --- | --- | --- | --- | --- | --- |
| d13C | 0.52** | 0.62** | 0.05 | 0.12 | 0.65* |
| SLA | 0.17 | 0.05 | 0.11 | 0.18 | 0.18 |
| PRI | 0.38* | 0.58** | 0.16 | 0.38* | 0.59* |
| NDVI | 0.08 | 0.001 | 0.005 | 0.01 | 0.18 |
| leaf area | 0.04 | 0.04 | 0.11 | 0.11 | 0.05 |
| wood density | 0.15 | 0.21 | 0.11 | 0.18 | 0.21 |
| NCONC | 0.35* | 0.24 | 0.02 | 0.09 | 0.35 |

Figure S1. Trait response to each climate variable within the general additive models (GAM). Solid lines are the response variables and the dashed lines are the 95% confidence intervals. Climate variables are as follows: P_MA_ = mean annual precipitation; P_DM_ = precipitation of the driest month; P_RANGE_ = precipitation variation; T_RANGE_ = temperature variation; T_MAX_ = maximum temperature of the warmest month; T_MA_ = mean annual temperature.
